# Renal resistive index as a marker of vascular damage in cardiovascular diseases

**DOI:** 10.1007/s11255-013-0528-6

**Published:** 2013-08-20

**Authors:** Arkadiusz Lubas, Grzegorz Kade, Stanisław Niemczyk

**Affiliations:** Department of Internal Diseases, Nephrology and Dialysis, Military Institute of Medicine, ul. Szaserów 128, 04-141 Warsaw 44, Poland

**Keywords:** Cardiovascular diseases, Hypertension, Vascular damage, Renal ultrasonography, Renal resistive index

## Abstract

The article presents changeability of renal resistive index (RRI) in various cardiovascular diseases and considers the usefulness of the marker and interpretational difficulties of the index. The values of RRI are not specific to an individual disease, but in a selected group of patients, it seems to be a perfect marker of cardiovasculorenal changes and a predictor of rapid loss of a renal function. The RRI usually does not reflect the vascular resistance, but is dependent on total and local vascular bed compliance changing with age, in the course of consecutive diseases and the influence of drugs. Under specific conditions, RRI appears to be a good marker of vascular damage. This review summarizes current concepts in RRI interpretation against the cardiovascular pathologies, focusing on the vascular damage association with regard to the complex nature of RRI value variability.

## Introduction

In recent years, an increasing number of reports appeared, which on the one hand created the renal resistive index (RI), as a promising marker for various types of vascular damage, but on the other hand made this parameter less specific or even inadequate to properties attributed to it. Renal RI (RRI) is defined as a ratio of the difference between maximum and minimum (end-diastolic) flow velocity to maximum flow velocity derived from the Doppler spectrum of intrarenal (segmental/interlobar) arteries. Normal RI values in adults are in the range of 0.47–0.70 with a difference between two kidneys of <5–8 % [1]. Originally, RI was proposed by Pourcelot [2] to define the resistance of blood flow in peripheral arteries and meant to be useful in carotid artery stenosis assessment. However, subsequent studies reported the usefulness of the RI evaluation, for example, in intrarenal, carotid, orbital and uterine arteries [3, 4]. Currently, resistive index measured in intrarenal segmental arteries is a well-known marker of renal vascular and interstitial damage, corresponding to an increased total cardiovascular risk [5–7]. The reproducibility and repeatability of RRI are in most cases sufficient or even very good, but depend on investigator experience and care in order to obtain high-quality data [8]. When measurements were taken by well-trained staff, intraobserver variability ranged from 2.07 to 5.1 %, while interobserver variability ranged from 3.61 to 6.2 % [6, 9, 10]. The intraobserver and interobserver differences between RRI values ranging from 0.02 to 0.04 should be considered as not significant.

Although an increasing number of reports demonstrate the suitability of RRI assessment for cardiovascular diseases (CVD), there is no agreement concerning the real nature of the RRI variation. Analyzing the achievements in this field, it can be concluded that, in stable conditions, the value of the RRI is mainly affected by age, pulse pressure (PP) and arterial stiffness, whose progression increases appropriately depending on the concomitant disease entity (e.g., diabetes, chronic kidney disease) [7, 11–17]. There are few experimental studies and observations denying the validity of identifying renal resistive index with renal vascular resistance. Thus, using an infusion of l-NG-monomethyl arginine (l-NMMA) with evaluation of para-aminohippuric acid and inulin clearance in 41 healthy subjects, Raff et al. [18] found neither baseline nor functional RRI correlation with the renal vascular resistance or renal perfusion. Nevertheless, infusion of l-NMMA and the inhibition of nitric oxide synthase in this study resulted in a maximal increase in RRI value—probably related to the increase in resistance (end-diastolic velocity = 0). In earlier experimental researches on artificial and animal models, results show dependence of RRI on: (1) the vascular compliance and resistance, with the relation of weakened vascular resistance dependency in time of vascular compliance decrease; (2) pulse pressure with a small dependence on nonphysiologic high vascular resistance; and (3) increased renal interstitial pressure, urinary tract and intra-abdominal pressure [19–21]. These studies show that regardless of the model, RRI value only slightly depends on the actual vascular resistance. Therefore, it seems that assessing the renal vascular resistance as RRI is misleading. Furthermore, clinical trials in patients with CVD confirmed significant and independent relation of RRI to age, PP and vascular stiffness without significant impact of renal function and pathological changes in the kidney biopsy, with the exception of atherosclerosis [5, 15]. Consequently, in the available literature common opinion was that RRI value is only a reflection of vascular bed atherosclerotic changes. This view is probably due to the observations of Akgul et al. [13] who showed RRI dependence on vascular alterations in patients after kidney transplantation. In this study, only PP independently determined the value of RRI. However, initially the damage of the transplanted kidney is usually smaller than the changes in patient’s vascular bed. For that reason, the hemodynamic impact of systemic changes has greater influence on RRI than changes in a vascular bed of the denervated kidney. However, in case of advanced nephropathy or acute rejection of a transplanted kidney, the situation changes and the impact of intrarenal damage on RRI value increases [22, 23].

In the present review, we discuss the usefulness of RRI against cardiovascular pathologies and focus on vascular damage association as well as the complex, especially dual, systemic and local nature of RRI value variability.

## Methods

A comprehensive search of PubMed was performed for trials published between January 2007 and December 2012. The search was performed by using the keywords: ‘renal resistive index’ and ‘renal resistance index,’ without any of the following words: ‘stone,’ ‘colic,’ ‘obstructions,’ ‘transplantation’ and ‘allograft’ occurring in the title/abstract. Exclusions were made because of the direct impact of elevated intrarenal pressure and disrupted autoregulation on RRI value. The reference lists of the gathered publications were reviewed for hypertension, obstructive sleep apnea, diabetes, acute and chronic renal failure.

## Results

We found 144 articles relating to ‘renal resistive index’ and 380 publications with ‘renal resistance index’ search. Considering the impact of cardiovascular diseases on RRI, we found in the last 6 years 27 studies relating to hypertension, 17 to chronic kidney failure, 16 to diabetes mellitus, eight concerning acute kidney injury in intensive care unit and three to obstructive sleep apnea.

## Discussion

### Hypertension

The majority of studies state a significant and independent association of RRI value with age, pulse wave velocity (PWV), PP and blood pressure (BP) [7, 11–14]. Due to the definition, the increase in RRI value is dependent on an increase in systolic BP, and/or on a reduction in diastolic BP, namely the increase in PP, which is the equivalent of vascular stiffness. Therefore, there is a significant dependence of RRI on age and PWV, thus indirectly on the cardiovascular risk. Kawai et al. [17] performing ambulatory blood pressure monitoring (ABPM) in 88 subjects with hypertension showed significantly higher RRI values in patients with a larger morning surge of BP (>32.5 mmHg), but the level of nocturnal systolic BP fall was not associated significantly with the values of RRI. In a different study by Kawai et al. [24], in a group of 143 patients with hypertension, high variability in outpatient visit-to-visit BP measurements was significantly and independently associated with higher values of RRI, and RRI proved to be a sensitive marker of BP variability. Moreover, in the study of 120 hypertensive patients, these authors demonstrated a significant correlation between daytime systolic BP variability, ambulatory arterial stiffness index (AASI) and RRI [25]. Examining 133 patients with hypertension, Hashimoto et al. [11] found a significant correlation of RRI with PWV, aortic PP and the albumin/creatinine ratio. Each increase in RRI by 0.1 caused a 5.4-fold increase in adjusted risk of albuminuria. The independent connection between RRI and markers of vascular-organ damage is showed in Florczak et al.’s [12] studies. These authors in a group of 223 patients with never-treated essential hypertension (EH) and a group of 95 healthy subjects found a significant and independent association of AASI and IMT with RRI. In a study based on 84 patients with refractory hypertension, Raff et al. [26] showed that the group of patients with RRI > 0.7 was characterized by significantly higher PWV, IMT and coronary artery calcification compared with patients with normal RRI. The association of RRI with signs of hypertensive target organ damage is visible despite mild to moderate chronic renal failure occurrence. In a group of 279 patients with primary hypertension, Derchi et al. [15] found a mild reduction in renal function (90 > eGFR ≥ 60 ml/min) in 96 patients. These participants had significantly higher signs of organ damage, including increased left ventricular mass, carotid intima-media thickness (IMT), systolic blood pressure, PP and higher RRI in comparison with patients without renal dysfunction. Despite kidney disease, RRI was still significantly dependent on age and PP. Examining 426 patients with treated EH, Doi et al. [27] found that the group with high RRI (for males ≥0.73, females ≥0.72) and eGFR < 60 ml/min/1.73 m^2^ had a significantly higher risk of the primary composite end points (nonfatal congestive heart failure, stroke, myocardial infarction, aortic dissection, death and end-stage renal failure requiring regular hemodialysis therapy) in comparison with the group with low RRI (for males <0.62 and females <0.67) and eGFR ≥ 60 ml/min/1.73 m^2^, during a mean follow-up of 3 years. Nevertheless, high prevalence of diabetes mellitus (28.9 %) and chronic renal failure limits these results for EH [28]. In a recently published study by Doi et al. [29], in a group of 288 patients with EH, higher RRI values were found in patients with preclinical target organ damage (presence of carotid wall thickening, left ventricular hypertrophy, albuminuria). The number of involved organs increased from lower to upper RRI tertile (for males ≥0.69 and females ≥0.72). However, in one of our studies the strict control of BP in patients with essential hypertension was associated with improvement in renal vascular reactivity measured in a dynamic assessment of RRI with captopril administration, suggesting a regression of vascular damage [30]. These studies show significant RRI dependence on the markers of cardiovascular damage in hypertension. As it turns out, possibilities of using the RRI in diagnosis of cardiovascular damage are even greater. In the analysis published by Ennezat et al. [31], based on a group of patients with hypertension and properly preserved ejection fraction, significantly higher RRI values were found in patients with clinical and echocardiographic markers of heart failure (HF) even after adjusting for renal function, BP and antihypertensive agents. High values of RRI significantly correlated with HF and were an independent predictor of poor outcome in these patients. Based on their observation, the authors demonstrate significant dependence of the RRI from vascular damage induced by HF and important predictive value of this indicator. However, this study is not that surprising, because previously Tedesco et al. [16] studying 566 patients with hypertension and preserved renal function found that age, PP, left ventricular mass index and IMT significantly and independently modify the value of the RRI. In this large-population study, RRI did not correlate with renal function. Regarding these studies, it seems that RRI is a reliable tool in monitoring organ damage state in the course of hypertension, despite mild to moderate chronic renal failure occurrence. Therefore, under specific conditions, RRI could be considered as a renal vascular damage index.

### Obstructive sleep apnea

The previous studies indicate that the increased risk of CVD in patients with obstructive sleep apnea (OSA) is associated with the secondary activation of the sympathetic nervous system and increased risk of developing hypertension and renal impairment. Buchner et al. [32] found significantly higher values of RRI in a group of 64 patients with OSA comparing to healthy subjects. After approximately 10 months of observation, decreased RRI values were found only in patients with effectively treated OSA. In this study, the presence of OSA was independent of hypertension, age, diabetes or kidney function risk factor for elevated RRI, which on the other hand turned out to be a sensitive marker of treatment effectiveness and reduction in sympathetic activity.

### Renovascular hypertension (renal artery stenosis)

A particular, potentially reversible, form of secondary hypertension is renovascular hypertension, caused by renal artery stenosis (RAS). Due to a possibility of hemodynamic renal artery assessment, RRI was used in RAS diagnosis. The difference in RRI values between kidneys >0.05 or ∆RRI > 8 % together with RRI < 0.45 suggests the presence of significant RAS [33, 34]. However, a successful renal artery revascularization does not always lead to improved BP control. Expanding the range of diagnostic and prognostic usefulness of RRI, Radermacher et al. [35] proposed application of this factor in predicting effects of RAS angioplasty. The researchers studying patients with RAS found a lack of balloon angioplasty efficacy in the form of better BP control and improvement of renal function in patients with RRI > 0.8. This study probably contributed to recommendations being made regarding contradictions to RAS revascularization in case of high values of RRI [36, 37]. However, the results of a later study by Zeller et al. [38] undermined the legitimacy of such recommendations. These authors, in a group of 241 patients with a severe stenosis (>70 %) of one of the renal arteries treated with angioplasty, found in 39 cases with RRI > 0.8 a significant improvement in the renal function and BP control. For this reason, it seems that the high value of the RRI cannot be considered as a sole marker of angioplasty failure. On the other hand, Cianci et al. [39] assessed 40 patients with RAS, 12 months after angioplasty with stent implantation, and presented that bilaterally elevated RRI (0.83 ± 0.2) with proteinuria is associated with the deterioration of kidney function after revascularization. In a recently published retrospective study, Yuksel et al. [40] in the group of 44 patients with RAS and RRI < 0.75 showed significant improvement in the renal function, BP control and need for antihypertensive drugs compared to baseline, after 1 year of follow-up from angioplasty and stenting. In a group of 29 patients with initial RRI > 0.75, significant progression of renal failure 11 months after revascularization was found. Currently, for patients with renovascular hypertension in the course of RAS, without urgent clinical symptoms, the continuation of drug therapy is considered [41–43]. In our study, RRI in dynamic captopril assessment proved to be a useful marker of changes in renal autoregulation and pharmacological treatment efficacy in patients with stenosis (>50 %) of one renal artery [44].

### Acute kidney injury in critically ill patients

Occurrence of acute kidney injury (AKI) in severe clinical state patients staying in intensive care units significantly increases the cardiovascular risk and worsens the patient’s prognosis. Reviewing recently published studies, there is an interesting work presented by Bossard et al. [45], who studied 65 patients after cardiac bypass graft surgery with risk factors for AKI and indicated that in the immediate postoperative period RRI value >0.74 predicted the occurrence of delayed AKI with a sensitivity of 85 % and a specificity of 94 %. Moreover, in a study of 51 critically ill patients hospitalized in an intensive care unit, Darmon et al. [46] showed that RRI > 0.795 identified patients with persistent AKI (>3 days) with a sensitivity of 92 % and a specificity of 85 %. On the basis of this study, the authors postulate usefulness of RRI assessment in predicting the reversibility of AKI in critically ill patients. Based on the prospective observational study of 96 septic, critically ill patients, Dewitte et al. [9] demonstrated median RRI 0.72 in patients without AKI, but in subjects with AKI, RRI was significantly higher (0.76). Recently, Shnell et al. [47], who studied 58 patients with severe sepsis or polytrauma, showed superiority of RRI on cystatin C in predicting AKI. In this study, RRI > 0.707 achieved in the first 12 h after admission to an intensive care unit was the only predictor of AKI stage 2 or 3 in the third day after admission. These findings show the complex nature of RRI changeability depending probably on an increase in renal vascular resistance in response to systemic vasodilatation as a compensatory mechanism maintaining GFR and, on the other hand, on renal damage [48]. In one recent study, based on a group of 20 patients with septic shock, the RRI proved to be as effective early predictor of AKI as NGAL (neutrophil gelatinase-associated lipocalin) [49].

### Chronic kidney disease (CKD)

Studies in small groups of patients showed a dependence of RRI on renal function and mainly atherosclerotic changes in histopathologic assessment of renal biopsy (RB) [5, 6, 50]. On the basis of RB in 58 CKD patients, Bigé et al. [51] demonstrated that RRI ≥ 0.65 was associated with severe interstitial fibrosis (>20 %), severe arteriosclerosis and decline of renal function in 18 months of follow-up. In addition, in the group of 202 Japanese patients diagnosed with RB, Hanamura et al. [52] found better renal survival in patients with RRI < 0.65, excellent response to steroid therapy when 0.65 ≤ RRI < 0.7, but resistance to steroids and a high risk of declining renal function in patients with RRI ≥ 0.7. These authors proposed RRI as a possible determinant of indication for steroid therapy.

However, it appears that RRI is not only a kidney organ damage marker, but it can also be considered as a predictor of onset and progression of CKD. It seems that in mild to moderate renal dysfunction, RRI is superior in prediction of chronic kidney disease progression and poor outcome to renal function estimation alone. This thesis could be supported by the evidence of significantly higher RRI values in patients with higher cardiovascular burden in comparison with those with lower RRI, despite equal creatinine concentration [16]. In a study of hypertensive patients by Okura et al. [53], in a stepwise regression analysis model using baseline RRI, age, PP, glycosylated hemoglobin, C-reactive protein, cystatin C and urinary albumin/creatinine ratio, only the baseline RRI was a marker of renal dysfunction in a 1-year observation. Parolini et al. [54] in a group of 86 subjects with nephropathies showed that patients with RRI > 0.7 were characterized by rapid progression of renal dysfunction and a decrease in eGFR > 50 % during 6 years of observation. Derchi et al. [15] reported 2.83-fold increased risk of mild renal dysfunction when RI ≥ 0.63. The ultimate confirmation of the RRI suitability in predicting renal function seems to be a study of Sugiura et al. [55]. In an observation of 281 patients with CKD, the authors demonstrated significantly higher incidence of worsening renal function in patients with RRI > 0.7. Renal resistive index >0.7 together with proteinuria (defined as >1.0 g/g creatinine), eGFR < 50 ml/min/1.73 m^2^ and high systolic BP (>140 mmHg) were independent predictors of renal function deterioration. What is more, in a 2-year follow-up period, the value of RRI > 0.7 proved to be as strong predictor of renal function worsening (hazard ratio (HR) 4.01; 95 % confidence interval (CI) 1.87–8.61; *p* < 0.001) as proteinuria and hypertension. Based on their observations, the authors recognize RRI as an independent risk factor for progression of CKD, suggesting the need of RRI assessment immediately after finding nephropathy. However, these findings are not so surprising, because previously Radermacher et al. [56] showed that high RRI > 0.8 identifies CKD patients at high risk of renal disease progression and death. In this study, RRI > 0.8 was better in indicating odds ratios for worsening renal function or death, in comparison with proteinuria >1 g/d or creatinine clearance <40 ml/min.

The vascular-related changeability of RRI value has a dual nature. On the one hand, it reflects changes in intrarenal environment, and on the other hand, it depends on systemic vascular conditions. Kawai et al. [17] in a group of 194 older patients (66 years) without RAS showed a significant and proportional RRI dependence on the stage of CKD. Furthermore, examining 140 patients with CKD, Heine et al. [7] stated a gradual increase in RRI value with progressing CKD. RRI value was independently associated with age, kidney function, the presence of diabetes and PP. Nevertheless, RRI was significantly higher in patients with type 2 diabetes in comparison with patients with nephropathy and without diabetes, which suggests additional independent influence of diabetes on RRI. These studies determine the confirmation of the impact of systemic as well as local intrarenal vascular changes on RRI value. On the one hand, the composite nature of RRI variability makes this parameter less organ specific, and on the other hand, it makes RRI a better predictor of cardiovasculorenal outcome than a single organ-specific parameter.

Although mild renal dysfunction expressed by diminished eGFR is a strong cardiovascular risk factor, it is a late marker of organ damage. Elevated RRI values identify high cardiovascular risk patients even before nephropathy occurred [16]. In a 1-year follow-up of 112 hypertensive patients without known nephropathy, the baseline RRI ≥ 0.7 was associated with significant worsening of renal function measured by serum cystatin C concentration [53]. Interestingly, during a follow-up period, creatinine and eGFR (modified MDRD formula) did not change in the group with RRI ≥ 0.7 as well as with RRI < 0.7. These data clearly support argumentation considering superiority of RRI in prediction of renal outcome even before nephropathy occurred. On the other hand, it is possible that dominance of RRI cardiovascular predictive properties would be diminished due to decreasing eGFR < 30 ml/min/1.73 m^2^, when influence of advanced local (intrarenal vascular and interstitial) alterations on RRI is greater than systemic.

### Diabetes

Diabetes as a metabolic disease with micro- and macroangiopathic complications significantly increases cardiovascular risk. In order to determine the usefulness of RRI in the diagnosis of subclinical vascular damage, Bruno et al. [57] studied a group of patients without albuminuria (32 patients with newly diagnosed type 2 diabetes and 49 patients with hypertension), assessing RRI before and after the administration of 25 μg of sublingual nitroglycerin and comparing with a group of 27 healthy subjects (age and sex matched). These authors found significantly higher RRI value in patients with diabetes when compared to the control group and the group with hypertension. However, the reduction in RRI after nitroglycerin administration was significantly lower in patients with diabetes when compared to those with hypertension and healthy subjects. These data indicate a greater vascular damage in patients with diabetes. The foregoing observations suggest the usefulness of dynamic RRI assessment in the diagnosis of subclinical and diabetogenic vascular damage, even before the onset of albuminuria. Recently, Liu et al. [58] examining 387 Chinese type 2 diabetic patients demonstrated significantly higher mean RRI values in those with microvascular diabetic complications, including nephropathy, retinopathy or sensory neuropathy, in comparison with subjects without complications. The RRI value >0.75 was associated with microvascular complications in diabetic patients. Another issue is the monitoring of vascular complications of diabetes. It turns out that, as in the case of hypertension, gradual vascular and organ damage in the course of type 2 diabetes is reflected in the value of RRI. It is confirmed by MacIsaac et al. [59], who in a group of 167 patients with type 2 diabetes, showed significantly higher RRI values in patients with echocardiographic markers of left ventricle diastolic dysfunction. It might be assumed that this relation stems from parallel organ damage of heart and kidneys in the course of type 2 diabetes.

It seems that the RI dependence of blood glucose levels may be specific to medium-sized arteries such as intrarenal and orbital. Confirmation of this hypothesis is the study of Afsar et al. [60], who stated that high RRI was independently associated with age, PP and insulin resistance measured by Homeostasis Model Assessment (HOMA) in a group of patients with newly diagnosed hypertension and type 2 diabetes. In addition, in the observation of 185 patients, Ohta et al. [3] reported a significant RRI correlation with blood glucose level. This dependence was not demonstrated for RI assessed in carotid arteries in the same patients. Recently, Basturk et al. [4] showed a significant positive correlation between intrarenal and orbital RI in patients with diabetes and nephropathy. Both intrarenal and orbital RIs were elevated in a group of 50 diabetic patients in comparison with 30 healthy subjects and were significantly higher in 53 patients with diabetes and nephropathy than in diabetes alone or controls.

On the other hand, it seems that the impact of diabetes on RRI is specific till the moment of a significant renal impairment. The argument confirming this hypothesis comes from the Kawai et al.’s [17] study, in which RRI was assessed in 70 patients with type 2 diabetes and in a group of 124 nondiabetic patients with hypertension and/or hyperlipidemia. These authors found significantly higher RRI values only in diabetic patients with one to three stages of CKD in comparison with those without diabetes, with an equivalent eGFR. In contrast, no differences in RRI were found between the groups with and without diabetes in patients with advanced stages of CKD (eGFR < 30 ml/min/1.73 m^2^). In other words, in the fourth and fifth stages of CKD, diabetes did not affect differences in RRI.

### Cardiovascular prediction properties

The data regarding prediction of all-cause and cardiovascular mortality by RRI are sparse. Heine et al. [7] reported independent association between RRI and Framingham risk score, IMT and PP. Moreover, in a study of large population of hypertensive patients, RRI was independently associated with age, IMT, LVMI and PP [16]. Ennezat et al. [31] showed a significant correlation of RRI and HF with preserved ejection fraction (HFpEF), and mean RRI values were strongly associated with HFpEF. In this study high values of RRI ≥ 0.82 were an independent predictor of poor outcome (death or hospitalization for HF decompensation) in patients with HFpEF (HR 1.06; 95 % CI 0.16–0.62; *p* = 0.007). In one available, prospective cohort study, after over 4-year observation of 726 elderly Americans, Pearce et al. [61] showed that RRI together with main renal artery peak systolic velocity (PSV) was significantly associated with all-cause mortality and cardiovascular event (hospitalized angina, congestive heart failure, myocardial infarction, coronary artery bypass grafting, stroke, transient ischemic attack), and only PSV proved to be a predictor of cardiovascular disease event. Nevertheless, these authors, in the course of approximately 5-year follow-up in 86 patients with atherosclerotic renovascular disease, showed that baseline (before revascularization) RRI ≥ 0.8 was the most powerful predictor of death (HR 6.7; 95 % CI 2.6–17; *p* < 0.001) [62]. Inconsistency in these results comes probably from different cardiovascular burden of examined patients (unselected elderly population vs. patients with RAS considered for revascularization). These data suggest growing significance of RRI in prediction of mortality due to cardiovascular risk rising. In contrast, diminished kidney function in CKD stage 3 (30 ≤ eGFR < 60 ml/min/1.73 m^2^) is associated with an increase in all-cause mortality with HR from nonsignificant up to 2.58 [63]. Similarly, as it is in RRI and cardiovascular burden, the risk of mortality in CKD rises due to diminishing renal function, more sharply in people with eGFR < 45 ml/min/1.73 m^2^. Although RRI correlates with renal function, and both are associated with higher cardiovascular and mortality risk, in cases of mild renal dysfunction and/or high cardiovascular burden not corresponding to renal function, RRI appears to be superior in cardiovascular risk assessment [7, 62].

## Conclusions

The values of RRI are not specific to an individual disease, but in a selected group of patients, it could be used as a good marker of cardiovasculorenal changes and a predictor of rapid loss of a renal function or even all-cause mortality. The RRI usually does not reflect the vascular resistance, but in CVD it is dependent on systemic and local vascular bed compliance changing with age, in the course of consecutive diseases and the influence of drugs. Depending on the investigated group of patients, RRI value is more closely related to the markers of vascular-organ damage in patients with CVD and diabetes (IMT, PP, PWV, albuminuria), or with the stage of renal impairment in nephropathies (GFR, proteinuria), and almost exclusively with the degree of intrarenal damage in acute or advanced chronic renal failure (acute urinary obstruction, parenchymal edema, arteriolosclerosis, interstitial fibrosis). Probably, for these reasons, in some studies, RRI appeared as a sensitive marker of EH and OSA treatment effectiveness or even a good indicator for steroid therapy in glomerulonephritis. On the basis of the previous studies, the point at which the RRI is more dependent on the intrarenal than systemic changes cannot be strictly determined. With the exclusion of acute clinical conditions modifying RRI value, eGFR 30 ml/min/1.73 m^2^ that corresponds to the limit of CKD between the third and fourth stages can be approximately considered a borderline between the dominance of systemic and local factors. However, in the study with a larger group of patients, the borderline of the systemic factors’ impact on RRI may need to be established on the other level of renal function. This problem requires further studies.
